# Poly[bis­(μ_4_-2,3,5,6-tetra­fluoro­benzene-1,4-di­carboxyl­ato-κ^4^ *O* ^1^:*O* ^1′^:*O* ^4^:*O* ^4′^)bis­(tetra­hydro­furan-κ*O*)dizinc]

**DOI:** 10.1107/S1600536813031887

**Published:** 2013-12-04

**Authors:** Sang Beom Choi, Young Ho Jhon, Nakeun Ko, Jin Kuk Yang

**Affiliations:** a Department of Chemistry, Soongsil University, 369 Sangdo-Ro, Dongjak-Gu, Seoul 156-743, Republic of Korea

## Abstract

The title compound, [Zn_2_(C_8_F_4_O_4_)_2_(C_4_H_8_O)_2_]_*n*_, has a three-dimensional metal-organic framework structure. The asymmetric unit consists of two Zn^II^ atoms, two tetrahydrofuran ligands, one 2,3,5,6-tetra­fluoro­benzene-1,4-di­carboxyl­ate ligand and two half 2,3,5,6-tetra­fluoro­benzene-1,4-di­carboxyl­ate ligands, which are completed by inversion symmetry. One Zn^II^ atom has a distorted trigonal–bipyramidal coordination geometry, while the other has a distorted octa­hedral geometry. Two independent tetra­hydro­furan ligands are each disordered over two sets of sites with occupancy ratios of 0.48 (4):0.52 (4) and 0.469 (17):0.531 (17).

## Related literature   

For general background of compounds with metal-organic framework structures, see: Yoon *et al.* (2007 ▶). For related crystal structures, see: Hulvey *et al.* (2011 ▶); Seidel *et al.* (2011 ▶); Yoon *et al.* (2007 ▶); Yu *et al.* (2011 ▶); Zheng *et al.* (2008 ▶).
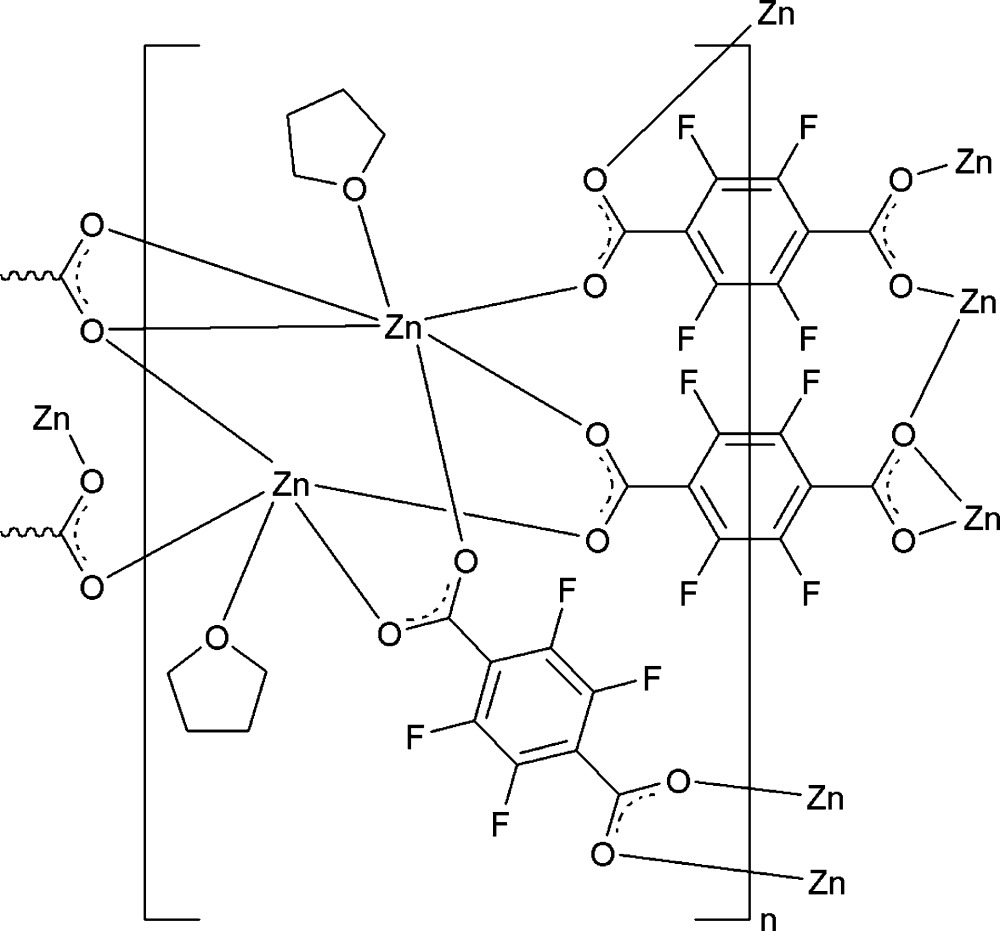


## Experimental   

### 

#### Crystal data  

[Zn_2_(C_8_F_4_O_4_)_2_(C_4_H_8_O)_2_]
*M* *_r_* = 747.11
Monoclinic, 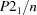

*a* = 11.9339 (8) Å
*b* = 12.4369 (9) Å
*c* = 17.9627 (12) Å
β = 104.051 (1)°
*V* = 2586.3 (3) Å^3^
*Z* = 4
Mo *K*α radiation
μ = 1.97 mm^−1^
*T* = 173 K
0.10 × 0.05 × 0.05 mm

#### Data collection  

SMART APEX CCD diffractometer
Absorption correction: multi-scan (*SADABS*; Sheldrick, 2003 ▶) *T*_min_ = 0.827, *T*_max_ = 0.908
16096 measured reflections
5980 independent reflections
2859 reflections with *I* > 2σ(*I*)
*R*_int_ = 0.111

#### Refinement  

*R*[*F* ^2^ > 2σ(*F* ^2^)] = 0.053
*wR*(*F* ^2^) = 0.139
*S* = 0.94
5980 reflections
444 parameters
81 restraints
H-atom parameters constrained
Δρ_max_ = 0.66 e Å^−3^
Δρ_min_ = −0.78 e Å^−3^

### 

Data collection: *SMART* (Bruker, 2007 ▶); cell refinement: *SAINT* (Bruker, 2007 ▶); data reduction: *SAINT*; program(s) used to solve structure: *SHELXTL* (Sheldrick, 2008 ▶); program(s) used to refine structure: *SHELXTL*; molecular graphics: *SHELXTL*; software used to prepare material for publication: *publCIF* (Westrip, 2010 ▶).

## Supplementary Material

Crystal structure: contains datablock(s) global, I. DOI: 10.1107/S1600536813031887/is5307sup1.cif

Structure factors: contains datablock(s) I. DOI: 10.1107/S1600536813031887/is5307Isup2.hkl

Additional supporting information:  crystallographic information; 3D view; checkCIF report

## Figures and Tables

**Fig. 1.**
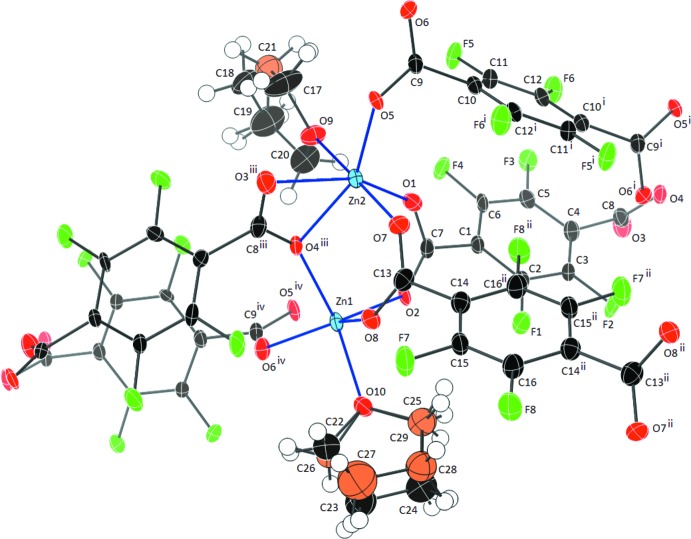
The coordination environment of the title compound is shown with the atomic numbering scheme. Displacement ellipsoids are drawn at 50% probability level. The atoms with the symmetry codes, (iii) and (iv) are not completely labeled for simplicity. [Symmetry codes: (i) 1 –x, –y, –z; (ii) 2 –x, –y, –z; (iii) 1/2 + x, 1/2 – y, 1/2 + z; (iv) 3/2 – x, 1/2 + y, 1/2 – z.]

**Fig. 2.**
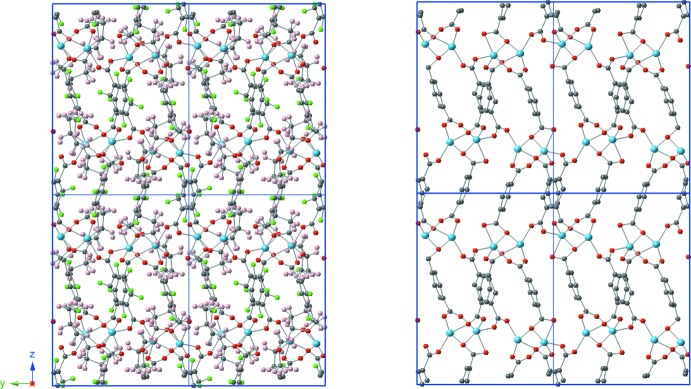
(left) A projection view along the a axis. (right) For comparison, a same packing diagram is displayed without THF molecules and F atoms.

**Fig. 3.**
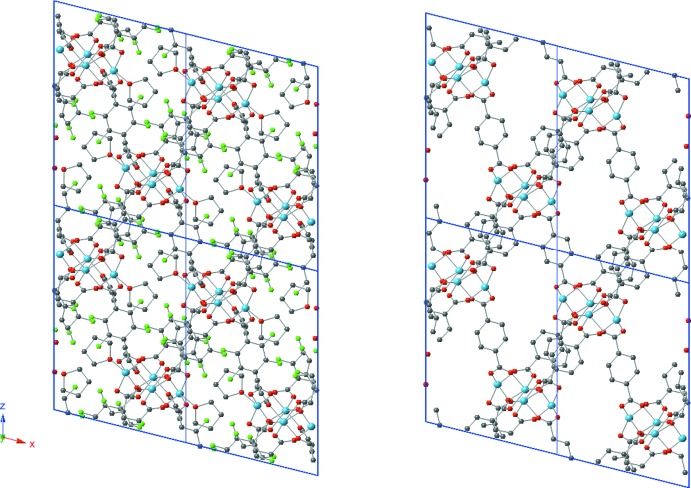
(left) A projection view along the b axis. (right) For comparison, a same packing diagram is displayed without THF molecules and F atoms.

## supplementary crystallographic information

### 1. Comment

We reported previously a porous metal-organic framework (MOF) composed of iron ions and the same ligand in the title compound (Yoon *et al.*, 2007). In the course of making a new MOF using zinc ion, the title compound was obtained as single crystals in hot tetrahydrofurane (THF). The title compound has a three-dimensional framework of which potential void space is filled with coordinated tetrahydrofuran (THF) molecules. Related crystal structures have been reported (Hulvey *et al.*, 2011; Seidel *et al.*, 2011; Yu *et al.*, 2011; Zheng *et al.*, 2008).

### 2. Experimental

A mixture of Zn(NO_3_)_2_.6H_2_O (56 mg, 0.19 mmol) and tetrafluoroterephthalic acid (15 mg, 0.063 mmol) was added to tetrahydrofuran (5.0 mL) in a Teflon-lined stainless steel autoclave. The mixture was heated at 85 °C for 24 h. Colorless needle crystals were collected and washed with neat *N*,*N*-dimethylformamide.

### 3. Refinement

Two THF molecules are statistically disordered over two sites, and their site occupancy factors were refined. Same distance and isotropic behaviour restraints (SADI and ISOR, respectively) were applied for the disordered molecules. H atoms were positions with idealized geometry (C—H = 0.99 Å) and allowed to ride with *U*_iso_(H) = 1.2*U*_eq_(C).

### Crystal data

**Table:**

[Zn_2_(C_8_F_4_O_4_)_2_(C_4_H_8_O)_2_]	*F*(000) = 1488
*M**_r_* = 747.11	*D*_x_ = 1.919 Mg m^−^^3^
Monoclinic, *P*2_1_/*n*	Mo *K*α radiation, λ = 0.71073 Å
Hall symbol: -P 2yn	Cell parameters from 2025 reflections
*a* = 11.9339 (8) Å	θ = 2.3–27.0°
*b* = 12.4369 (9) Å	µ = 1.97 mm^−^^1^
*c* = 17.9627 (12) Å	*T* = 173 K
β = 104.051 (1)°	Needle, colorless
*V* = 2586.3 (3) Å^3^	0.10 × 0.05 × 0.05 mm
*Z* = 4	

### Data collection

**Table:**

SMART APEX CCD diffractometer	5980 independent reflections
Radiation source: fine-focus sealed tube	2859 reflections with *I* > 2σ(*I*)
Graphite monochromator	*R*_int_ = 0.111
phi and ω scans	θ_max_ = 27.9°, θ_min_ = 1.9°
Absorption correction: multi-scan (*SADABS*; Sheldrick, 2003)	*h* = −15→14
*T*_min_ = 0.827, *T*_max_ = 0.908	*k* = −16→13
16096 measured reflections	*l* = −23→23

### Refinement

**Table:**

Refinement on *F*^2^	81 restraints
Least-squares matrix: full	H-atom parameters constrained
*R*[*F*^2^ > 2σ(*F*^2^)] = 0.053	*w* = 1/[σ^2^(*F*_o_^2^) + (0.0519*P*)^2^] where *P* = (*F*_o_^2^ + 2*F*_c_^2^)/3
*wR*(*F*^2^) = 0.139	(Δ/σ)_max_ < 0.001
*S* = 0.94	Δρ_max_ = 0.66 e Å^−^^3^
5980 reflections	Δρ_min_ = −0.78 e Å^−^^3^
444 parameters	

### Special details

**Table:**

Geometry. Geometry. All e.s.d.'s (except the e.s.d. in the dihedral angle between two l.s. planes) are estimated using the full covariance matrix. The cell e.s.d.'s are taken into account individually in the estimation of e.s.d.'s in distances, angles and torsion angles; correlations between e.s.d.'s in cell parameters are only used when they are defined by crystal symmetry. An approximate (isotropic) treatment of cell e.s.d.'s is used for estimating e.s.d.'s involving l.s. planes.Refinement. Refinement of F^2^ against ALL reflections. The weighted *R*-factor *wR* and goodness of fit S are based on F^2^, conventional *R*-factors *R* are based on F, with F set to zero for negative F^2^. The threshold expression of F^2^ > 2sigma(F^2^) is used only for calculating *R*-factors(gt) *etc*. and is not relevant to the choice of reflections for refinement. *R*-factors based on F^2^ are statistically about twice as large as those based on F, and R– factors based on ALL data will be even larger.

### Fractional atomic coordinates and isotropic or equivalent isotropic displacement parameters (Å^2^)

**Table:**

	*x*	*y*	*z*	*U*_iso_*/*U*_eq_	Occ. (<1)
Zn1	0.95391 (6)	0.25834 (6)	0.23174 (4)	0.0190 (2)	
Zn2	0.74360 (6)	0.06295 (6)	0.22126 (4)	0.0197 (2)	
F1	0.7946 (3)	0.3132 (4)	−0.0193 (2)	0.0360 (11)	
F2	0.6468 (3)	0.3797 (4)	−0.14747 (19)	0.0380 (11)	
F3	0.3476 (3)	0.3893 (3)	−0.0176 (2)	0.0343 (10)	
F4	0.4890 (3)	0.3084 (3)	0.1070 (2)	0.0350 (11)	
F5	0.3829 (3)	0.0673 (3)	0.10356 (19)	0.0319 (10)	
F6	0.3318 (3)	0.1509 (3)	−0.0385 (2)	0.0314 (10)	
F7	1.1600 (3)	−0.0283 (3)	0.1343 (2)	0.0380 (11)	
F8	1.2137 (3)	−0.0770 (3)	0.0024 (2)	0.0363 (11)	
O1	0.6808 (4)	0.1774 (4)	0.1436 (2)	0.0294 (12)	
O2	0.8233 (3)	0.2972 (4)	0.1440 (2)	0.0231 (11)	
O3	0.3584 (4)	0.5058 (4)	−0.1657 (2)	0.0315 (12)	
O4	0.3716 (3)	0.3404 (4)	−0.2065 (2)	0.0211 (10)	
O5	0.6454 (3)	−0.0690 (4)	0.2015 (2)	0.0236 (11)	
O6	0.4829 (3)	−0.1595 (4)	0.1690 (2)	0.0217 (10)	
O7	0.8535 (4)	0.0103 (4)	0.1569 (2)	0.0308 (12)	
O8	1.0048 (4)	0.1237 (4)	0.1836 (2)	0.0264 (11)	
C1	0.6473 (5)	0.3081 (5)	0.0487 (3)	0.0188 (14)	
C2	0.6838 (5)	0.3309 (6)	−0.0176 (3)	0.0222 (16)	
C3	0.6081 (5)	0.3672 (5)	−0.0829 (3)	0.0223 (15)	
C4	0.4933 (5)	0.3872 (5)	−0.0855 (3)	0.0215 (15)	
C5	0.4593 (5)	0.3698 (5)	−0.0191 (4)	0.0209 (15)	
C6	0.5307 (5)	0.3282 (5)	0.0456 (3)	0.0208 (15)	
C7	0.7248 (5)	0.2574 (6)	0.1181 (3)	0.0203 (15)	
C8	0.4033 (6)	0.4161 (5)	−0.1576 (4)	0.0224 (16)	
C9	0.5536 (5)	−0.0941 (5)	0.1541 (3)	0.0187 (15)	
C10	0.5264 (5)	−0.0450 (5)	0.0742 (3)	0.0197 (15)	
C11	0.4409 (5)	0.0323 (5)	0.0528 (3)	0.0195 (15)	
C12	0.4157 (5)	0.0753 (5)	−0.0206 (3)	0.0171 (14)	
C13	0.9406 (6)	0.0548 (6)	0.1450 (4)	0.0255 (16)	
C14	0.9707 (6)	0.0254 (5)	0.0699 (4)	0.0233 (16)	
C15	1.0804 (5)	−0.0119 (6)	0.0676 (3)	0.0236 (16)	
C16	1.1077 (6)	−0.0371 (6)	0.0006 (4)	0.0256 (16)	
O9	0.6305 (4)	0.1245 (4)	0.2867 (3)	0.0305 (12)	
O10	1.0752 (4)	0.3383 (4)	0.1859 (2)	0.0315 (12)	
C17	0.5732 (8)	0.0626 (7)	0.3327 (5)	0.062 (3)	
H17A	0.6241	0.0039	0.3585	0.075*	0.48 (4)
H17B	0.5020	0.0304	0.3004	0.075*	0.48 (4)
H17C	0.6299	0.0316	0.3771	0.075*	0.52 (4)
H17D	0.5294	0.0031	0.3023	0.075*	0.52 (4)
C18	0.544 (3)	0.1357 (7)	0.3906 (13)	0.046 (8)	0.48 (4)
H18A	0.6024	0.1297	0.4401	0.055*	0.48 (4)
H18B	0.4673	0.1178	0.3990	0.055*	0.48 (4)
C21	0.4935 (15)	0.1368 (7)	0.3593 (17)	0.044 (6)	0.52 (4)
H21A	0.4879	0.1175	0.4117	0.053*	0.52 (4)
H21B	0.4153	0.1338	0.3245	0.053*	0.52 (4)
C19	0.5440 (8)	0.2459 (7)	0.3585 (5)	0.065 (3)	
H19A	0.5796	0.2976	0.3993	0.078*	0.48 (4)
H19B	0.4641	0.2696	0.3348	0.078*	0.48 (4)
H19C	0.5939	0.2643	0.4095	0.078*	0.52 (4)
H19D	0.4826	0.3009	0.3440	0.078*	0.52 (4)
C20	0.6139 (7)	0.2383 (6)	0.2990 (5)	0.046 (2)	
H20A	0.5722	0.2730	0.2506	0.055*	
H20B	0.6894	0.2746	0.3175	0.055*	
C22	1.0721 (13)	0.337 (3)	0.1044 (4)	0.036 (13)	0.531 (17)
H22A	1.0110	0.3858	0.0757	0.044*	0.531 (17)
H22B	1.0571	0.2637	0.0832	0.044*	0.531 (17)
C23	1.1889 (11)	0.3754 (18)	0.0989 (8)	0.059 (6)	0.531 (17)
H23A	1.2177	0.3313	0.0615	0.071*	0.531 (17)
H23B	1.1854	0.4514	0.0822	0.071*	0.531 (17)
C24	1.2664 (13)	0.3637 (18)	0.1776 (7)	0.057 (6)	0.531 (17)
H24A	1.3007	0.4340	0.1964	0.069*	0.531 (17)
H24B	1.3298	0.3127	0.1767	0.069*	0.531 (17)
C25	1.1943 (7)	0.3222 (17)	0.2292 (8)	0.044 (5)	0.531 (17)
H25A	1.2099	0.2450	0.2408	0.053*	0.531 (17)
H25B	1.2102	0.3629	0.2780	0.053*	0.531 (17)
C26	1.1923 (7)	0.3668 (15)	0.2270 (8)	0.028 (5)	0.469 (17)
H26A	1.2082	0.3472	0.2820	0.034*	0.469 (17)
H26B	1.2085	0.4441	0.2219	0.034*	0.469 (17)
C27	1.2578 (19)	0.297 (2)	0.1839 (9)	0.085 (8)	0.469 (17)
H27A	1.3343	0.3295	0.1862	0.102*	0.469 (17)
H27B	1.2702	0.2253	0.2082	0.102*	0.469 (17)
C28	1.1923 (13)	0.2859 (18)	0.1020 (9)	0.050 (6)	0.469 (17)
H28A	1.1890	0.2100	0.0849	0.060*	0.469 (17)
H28B	1.2265	0.3305	0.0675	0.060*	0.469 (17)
C29	1.0754 (15)	0.326 (3)	0.1053 (5)	0.046 (18)	0.469 (17)
H29A	1.0597	0.3965	0.0786	0.056*	0.469 (17)
H29B	1.0149	0.2749	0.0799	0.056*	0.469 (17)

### Atomic displacement parameters (Å^2^)

**Table:**

	*U*^11^	*U*^22^	*U*^33^	*U*^12^	*U*^13^	*U*^23^
Zn1	0.0188 (4)	0.0257 (5)	0.0107 (3)	−0.0033 (3)	0.0004 (3)	−0.0011 (4)
Zn2	0.0196 (4)	0.0255 (5)	0.0122 (4)	0.0005 (4)	0.0004 (3)	−0.0040 (4)
F1	0.017 (2)	0.068 (3)	0.022 (2)	0.012 (2)	0.0021 (17)	0.008 (2)
F2	0.034 (2)	0.067 (3)	0.0146 (19)	0.015 (2)	0.0089 (18)	0.004 (2)
F3	0.017 (2)	0.058 (3)	0.026 (2)	0.006 (2)	0.0027 (17)	0.009 (2)
F4	0.026 (2)	0.061 (3)	0.018 (2)	0.012 (2)	0.0071 (18)	0.003 (2)
F5	0.035 (2)	0.047 (3)	0.0158 (18)	0.0017 (19)	0.0109 (17)	0.012 (2)
F6	0.038 (2)	0.034 (3)	0.021 (2)	0.0058 (18)	0.0038 (18)	0.0145 (19)
F7	0.033 (2)	0.059 (3)	0.019 (2)	−0.002 (2)	0.0005 (18)	0.010 (2)
F8	0.022 (2)	0.057 (3)	0.029 (2)	−0.005 (2)	0.0049 (18)	0.009 (2)
O1	0.022 (3)	0.036 (3)	0.025 (3)	0.007 (2)	−0.003 (2)	−0.006 (2)
O2	0.019 (2)	0.031 (3)	0.013 (2)	0.003 (2)	−0.0070 (19)	−0.002 (2)
O3	0.041 (3)	0.026 (3)	0.023 (3)	−0.001 (2)	0.001 (2)	0.008 (2)
O4	0.021 (2)	0.030 (3)	0.011 (2)	0.002 (2)	0.0017 (19)	0.001 (2)
O5	0.025 (2)	0.027 (3)	0.013 (2)	0.003 (2)	−0.0051 (19)	−0.007 (2)
O6	0.023 (2)	0.026 (3)	0.016 (2)	0.006 (2)	0.0026 (19)	−0.005 (2)
O7	0.032 (3)	0.039 (3)	0.026 (3)	−0.010 (2)	0.016 (2)	−0.012 (2)
O8	0.025 (3)	0.032 (3)	0.022 (2)	−0.014 (2)	0.007 (2)	−0.005 (2)
C1	0.018 (3)	0.025 (4)	0.012 (3)	0.001 (3)	0.000 (3)	0.001 (3)
C2	0.018 (3)	0.033 (4)	0.013 (3)	−0.002 (3)	0.001 (3)	0.000 (3)
C3	0.026 (4)	0.028 (4)	0.011 (3)	−0.002 (3)	0.003 (3)	−0.006 (3)
C4	0.025 (4)	0.023 (4)	0.016 (3)	−0.004 (3)	0.003 (3)	0.005 (3)
C5	0.017 (3)	0.023 (4)	0.020 (3)	−0.002 (3)	0.000 (3)	0.004 (3)
C6	0.018 (3)	0.032 (4)	0.010 (3)	0.002 (3)	−0.003 (3)	0.001 (3)
C7	0.019 (3)	0.029 (4)	0.012 (3)	−0.002 (3)	0.003 (3)	0.004 (3)
C8	0.030 (4)	0.019 (4)	0.017 (3)	−0.005 (3)	0.003 (3)	−0.003 (3)
C9	0.025 (4)	0.016 (4)	0.013 (3)	0.000 (3)	0.001 (3)	0.000 (3)
C10	0.017 (3)	0.026 (4)	0.014 (3)	−0.001 (3)	0.000 (3)	−0.002 (3)
C11	0.022 (3)	0.021 (4)	0.014 (3)	0.000 (3)	0.003 (3)	0.003 (3)
C12	0.013 (3)	0.019 (4)	0.016 (3)	−0.002 (3)	−0.002 (3)	0.004 (3)
C13	0.031 (4)	0.025 (4)	0.023 (4)	−0.005 (3)	0.012 (3)	0.001 (3)
C14	0.028 (4)	0.021 (4)	0.020 (3)	−0.003 (3)	0.006 (3)	0.001 (3)
C15	0.024 (4)	0.028 (4)	0.016 (3)	−0.001 (3)	0.001 (3)	0.002 (3)
C16	0.025 (4)	0.031 (5)	0.022 (4)	−0.006 (3)	0.007 (3)	0.003 (3)
O9	0.031 (3)	0.026 (3)	0.040 (3)	−0.002 (2)	0.019 (2)	−0.002 (2)
O10	0.024 (3)	0.045 (3)	0.025 (3)	−0.010 (2)	0.007 (2)	−0.009 (2)
C17	0.078 (7)	0.047 (6)	0.084 (7)	0.001 (5)	0.061 (6)	−0.003 (5)
C18	0.062 (18)	0.048 (13)	0.038 (12)	0.003 (9)	0.031 (13)	−0.006 (10)
C21	0.045 (6)	0.045 (6)	0.045 (6)	−0.001 (2)	0.012 (2)	−0.001 (2)
C19	0.086 (7)	0.050 (7)	0.073 (6)	−0.008 (6)	0.046 (6)	−0.003 (6)
C20	0.059 (5)	0.030 (5)	0.055 (5)	−0.003 (4)	0.026 (4)	0.008 (4)
C22	0.037 (13)	0.037 (13)	0.036 (13)	0.000 (2)	0.011 (4)	−0.001 (2)
C23	0.059 (6)	0.061 (6)	0.059 (6)	0.001 (2)	0.017 (2)	−0.001 (2)
C24	0.056 (6)	0.059 (6)	0.058 (6)	0.002 (2)	0.016 (2)	0.000 (2)
C25	0.043 (5)	0.045 (5)	0.044 (5)	0.000 (2)	0.011 (2)	−0.001 (2)
C26	0.027 (5)	0.028 (5)	0.029 (5)	0.000 (2)	0.007 (2)	−0.001 (2)
C27	0.084 (9)	0.085 (9)	0.085 (9)	−0.001 (2)	0.021 (3)	0.001 (2)
C28	0.050 (6)	0.051 (6)	0.050 (6)	−0.001 (2)	0.014 (2)	0.001 (2)
C29	0.047 (18)	0.047 (18)	0.046 (18)	0.000 (2)	0.013 (5)	−0.001 (2)

### Geometric parameters (Å, º)

**Table:**

Zn1—O2	1.988 (4)	O9—C20	1.454 (8)
Zn1—O6^i^	2.034 (4)	O10—C22	1.455 (6)
Zn1—O8	2.043 (4)	O10—C29	1.456 (6)
Zn1—O4^ii^	2.058 (4)	O10—C26	1.457 (6)
Zn1—O10	2.084 (4)	O10—C25	1.459 (6)
Zn2—O5	1.998 (4)	C17—C18	1.484 (9)
Zn2—O1	2.007 (5)	C17—C21	1.485 (9)
Zn2—O7	2.055 (4)	C17—H17A	0.9900
Zn2—O4^ii^	2.122 (4)	C17—H17B	0.9900
Zn2—O9	2.136 (4)	C17—H17C	0.9900
Zn2—O3^ii^	2.319 (4)	C17—H17D	0.9900
F1—C2	1.348 (7)	C18—C19	1.487 (9)
F2—C3	1.357 (7)	C18—H18A	0.9900
F3—C5	1.362 (7)	C18—H18B	0.9900
F4—C6	1.338 (7)	C21—C19	1.485 (9)
F5—C11	1.344 (7)	C21—H21A	0.9900
F6—C12	1.354 (7)	C21—H21B	0.9900
F7—C15	1.352 (6)	C19—C20	1.511 (10)
F8—C16	1.352 (7)	C19—H19A	0.9900
O1—C7	1.262 (7)	C19—H19B	0.9900
O2—C7	1.256 (7)	C19—H19C	0.9900
O3—C8	1.231 (7)	C19—H19D	0.9900
O4—C8	1.280 (7)	C20—H20A	0.9900
O5—C9	1.253 (7)	C20—H20B	0.9900
O6—C9	1.246 (7)	C22—C23	1.498 (8)
O7—C13	1.241 (7)	C22—H22A	0.9900
O8—C13	1.243 (7)	C22—H22B	0.9900
C1—C2	1.393 (8)	C23—C24	1.497 (8)
C1—C6	1.402 (8)	C23—H23A	0.9900
C1—C7	1.499 (8)	C23—H23B	0.9900
C2—C3	1.372 (8)	C24—C25	1.501 (8)
C3—C4	1.382 (8)	C24—H24A	0.9900
C4—C5	1.366 (8)	C24—H24B	0.9900
C4—C8	1.512 (8)	C25—H25A	0.9900
C5—C6	1.365 (8)	C25—H25B	0.9900
C9—C10	1.521 (8)	C26—C27	1.501 (8)
C10—C12^iii^	1.365 (8)	C26—H26A	0.9900
C10—C11	1.387 (8)	C26—H26B	0.9900
C11—C12	1.388 (8)	C27—C28	1.496 (8)
C12—C10^iii^	1.365 (8)	C27—H27A	0.9900
C13—C14	1.522 (8)	C27—H27B	0.9900
C14—C16^iv^	1.388 (8)	C28—C29	1.498 (8)
C14—C15	1.398 (8)	C28—H28A	0.9900
C15—C16	1.358 (8)	C28—H28B	0.9900
C16—C14^iv^	1.388 (8)	C29—H29A	0.9900
O9—C17	1.420 (9)	C29—H29B	0.9900
			
O2—Zn1—O6^i^	128.65 (18)	C25—O10—Zn1	113.8 (6)
O2—Zn1—O8	96.92 (18)	O9—C17—C18	107.7 (8)
O6^i^—Zn1—O8	134.23 (17)	O9—C17—C21	106.6 (9)
O2—Zn1—O4^ii^	100.81 (17)	O9—C17—H17A	110.2
O6^i^—Zn1—O4^ii^	87.19 (16)	C18—C17—H17A	110.2
O8—Zn1—O4^ii^	88.21 (18)	O9—C17—H17B	110.2
O2—Zn1—O10	93.25 (17)	C18—C17—H17B	110.2
O6^i^—Zn1—O10	87.80 (17)	H17A—C17—H17B	108.5
O8—Zn1—O10	85.27 (18)	O9—C17—H17C	110.4
O4^ii^—Zn1—O10	165.12 (17)	C21—C17—H17C	110.4
O5—Zn2—O1	110.81 (17)	O9—C17—H17D	110.4
O5—Zn2—O7	93.75 (18)	C21—C17—H17D	110.4
O1—Zn2—O7	90.97 (19)	H17C—C17—H17D	108.6
O5—Zn2—O4^ii^	151.45 (16)	C17—C18—C19	105.7 (9)
O1—Zn2—O4^ii^	96.62 (18)	C17—C18—H18A	110.6
O7—Zn2—O4^ii^	93.52 (16)	C19—C18—H18A	110.6
O5—Zn2—O9	88.31 (18)	C17—C18—H18B	110.6
O1—Zn2—O9	87.05 (18)	C19—C18—H18B	110.6
O7—Zn2—O9	177.55 (19)	H18A—C18—H18B	108.7
O4^ii^—Zn2—O9	85.28 (16)	C17—C21—C19	105.8 (9)
O5—Zn2—O3^ii^	92.81 (16)	C17—C21—H21A	110.6
O1—Zn2—O3^ii^	155.73 (18)	C19—C21—H21A	110.6
O7—Zn2—O3^ii^	93.03 (17)	C17—C21—H21B	110.6
O4^ii^—Zn2—O3^ii^	59.25 (17)	C19—C21—H21B	110.6
O9—Zn2—O3^ii^	88.19 (17)	H21A—C21—H21B	108.7
C7—O1—Zn2	133.9 (4)	C21—C19—C20	104.2 (9)
C7—O2—Zn1	132.9 (4)	C18—C19—C20	105.5 (8)
C8—O3—Zn2^v^	85.2 (4)	C18—C19—H19A	110.6
C8—O4—Zn1^v^	135.1 (4)	C20—C19—H19A	110.6
C8—O4—Zn2^v^	92.9 (4)	C18—C19—H19B	110.6
Zn1^v^—O4—Zn2^v^	112.04 (18)	C20—C19—H19B	110.6
C9—O5—Zn2	134.8 (4)	H19A—C19—H19B	108.8
C9—O6—Zn1^vi^	113.0 (4)	C21—C19—H19C	110.9
C13—O7—Zn2	129.1 (4)	C20—C19—H19C	110.9
C13—O8—Zn1	126.5 (4)	C21—C19—H19D	110.9
C2—C1—C6	116.3 (5)	C20—C19—H19D	110.9
C2—C1—C7	122.6 (5)	H19C—C19—H19D	108.9
C6—C1—C7	120.9 (5)	O9—C20—C19	106.7 (6)
F1—C2—C3	118.8 (5)	O9—C20—H20A	110.4
F1—C2—C1	119.8 (5)	C19—C20—H20A	110.4
C3—C2—C1	121.3 (6)	O9—C20—H20B	110.4
F2—C3—C2	118.5 (6)	C19—C20—H20B	110.4
F2—C3—C4	119.4 (5)	H20A—C20—H20B	108.6
C2—C3—C4	122.0 (6)	O10—C22—C23	105.5 (10)
C5—C4—C3	116.4 (6)	O10—C22—H22A	110.6
C5—C4—C8	119.1 (5)	C23—C22—H22A	110.6
C3—C4—C8	124.3 (6)	O10—C22—H22B	110.6
F3—C5—C4	119.2 (5)	C23—C22—H22B	110.6
F3—C5—C6	117.5 (6)	H22A—C22—H22B	108.8
C4—C5—C6	123.1 (6)	C24—C23—C22	106.1 (12)
F4—C6—C5	119.4 (5)	C24—C23—H23A	110.5
F4—C6—C1	119.9 (5)	C22—C23—H23A	110.5
C5—C6—C1	120.6 (6)	C24—C23—H23B	110.5
O2—C7—O1	127.7 (6)	C22—C23—H23B	110.5
O2—C7—C1	118.4 (6)	H23A—C23—H23B	108.7
O1—C7—C1	114.0 (5)	C23—C24—C25	107.5 (11)
O3—C8—O4	122.6 (6)	C23—C24—H24A	110.2
O3—C8—C4	121.0 (6)	C25—C24—H24A	110.2
O4—C8—C4	116.2 (6)	C23—C24—H24B	110.2
O6—C9—O5	122.8 (5)	C25—C24—H24B	110.2
O6—C9—C10	117.7 (5)	H24A—C24—H24B	108.5
O5—C9—C10	119.5 (6)	O10—C25—C24	104.7 (9)
C12^iii^—C10—C11	117.4 (6)	O10—C25—H25A	110.8
C12^iii^—C10—C9	121.7 (6)	C24—C25—H25A	110.8
C11—C10—C9	120.8 (6)	O10—C25—H25B	110.8
F5—C11—C12	119.5 (5)	C24—C25—H25B	110.8
F5—C11—C10	120.2 (5)	H25A—C25—H25B	108.9
C12—C11—C10	120.3 (6)	O10—C26—C27	98.8 (11)
F6—C12—C10^iii^	119.7 (5)	O10—C26—H26A	112.0
F6—C12—C11	118.1 (5)	C27—C26—H26A	112.0
C10^iii^—C12—C11	122.2 (6)	O10—C26—H26B	112.0
O7—C13—O8	129.8 (6)	C27—C26—H26B	112.0
O7—C13—C14	115.7 (6)	H26A—C26—H26B	109.7
O8—C13—C14	114.5 (6)	C28—C27—C26	110.3 (14)
C16^iv^—C14—C15	115.9 (6)	C28—C27—H27A	109.6
C16^iv^—C14—C13	122.1 (6)	C26—C27—H27A	109.6
C15—C14—C13	122.0 (6)	C28—C27—H27B	109.6
F7—C15—C16	118.8 (6)	C26—C27—H27B	109.6
F7—C15—C14	119.0 (6)	H27A—C27—H27B	108.1
C16—C15—C14	122.1 (6)	C27—C28—C29	101.5 (14)
F8—C16—C15	119.3 (6)	C27—C28—H28A	111.5
F8—C16—C14^iv^	118.7 (6)	C29—C28—H28A	111.5
C15—C16—C14^iv^	122.0 (6)	C27—C28—H28B	111.5
C17—O9—C20	109.7 (5)	C29—C28—H28B	111.5
C17—O9—Zn2	125.7 (4)	H28A—C28—H28B	109.3
C20—O9—Zn2	124.1 (4)	O10—C29—C28	107.4 (11)
C29—O10—C26	107.2 (11)	O10—C29—H29A	110.2
C22—O10—C25	108.6 (9)	C28—C29—H29A	110.2
C22—O10—Zn1	122.9 (8)	O10—C29—H29B	110.2
C29—O10—Zn1	120.9 (12)	C28—C29—H29B	110.2
C26—O10—Zn1	126.0 (8)	H29A—C29—H29B	108.5
			
C6—C1—C2—F1	−179.6 (6)	F5—C11—C12—C10^iii^	178.3 (6)
C7—C1—C2—F1	−4.0 (10)	C10—C11—C12—C10^iii^	−0.6 (11)
C6—C1—C2—C3	−2.3 (10)	Zn2—O7—C13—O8	23.7 (11)
C7—C1—C2—C3	173.3 (6)	Zn2—O7—C13—C14	−152.8 (4)
F1—C2—C3—F2	2.6 (10)	Zn1—O8—C13—O7	−45.1 (11)
C1—C2—C3—F2	−174.8 (6)	Zn1—O8—C13—C14	131.4 (5)
F1—C2—C3—C4	−180.0 (6)	O7—C13—C14—C16^iv^	55.4 (9)
C1—C2—C3—C4	2.7 (11)	O8—C13—C14—C16^iv^	−121.6 (7)
F2—C3—C4—C5	178.0 (6)	O7—C13—C14—C15	−126.7 (7)
C2—C3—C4—C5	0.6 (10)	O8—C13—C14—C15	56.2 (9)
F2—C3—C4—C8	4.0 (10)	C16^iv^—C14—C15—F7	−178.0 (6)
C2—C3—C4—C8	−173.4 (6)	C13—C14—C15—F7	4.0 (10)
C3—C4—C5—F3	179.2 (6)	C16^iv^—C14—C15—C16	−1.4 (11)
C8—C4—C5—F3	−6.4 (10)	C13—C14—C15—C16	−179.3 (7)
C3—C4—C5—C6	−4.3 (10)	F7—C15—C16—F8	−0.6 (10)
C8—C4—C5—C6	170.0 (6)	C14—C15—C16—F8	−177.3 (6)
F3—C5—C6—F4	0.0 (9)	F7—C15—C16—C14^iv^	178.1 (6)
C4—C5—C6—F4	−176.5 (6)	C14—C15—C16—C14^iv^	1.4 (12)
F3—C5—C6—C1	−178.7 (6)	C20—O9—C17—C18	−14.5 (17)
C4—C5—C6—C1	4.8 (11)	Zn2—O9—C17—C18	157.5 (15)
C2—C1—C6—F4	−180.0 (6)	C20—O9—C17—C21	14.8 (14)
C7—C1—C6—F4	4.3 (10)	Zn2—O9—C17—C21	−173.2 (11)
C2—C1—C6—C5	−1.3 (10)	O9—C17—C18—C19	22 (3)
C7—C1—C6—C5	−177.0 (6)	C21—C17—C18—C19	−70.7 (8)
Zn1—O2—C7—O1	0.7 (10)	O9—C17—C21—C19	−26 (2)
Zn1—O2—C7—C1	−179.3 (4)	C18—C17—C21—C19	70.9 (8)
Zn2—O1—C7—O2	−11.3 (11)	C17—C21—C19—C18	−70.7 (8)
Zn2—O1—C7—C1	168.7 (4)	C17—C21—C19—C20	26 (2)
C2—C1—C7—O2	49.9 (9)	C17—C18—C19—C21	70.8 (8)
C6—C1—C7—O2	−134.7 (7)	C17—C18—C19—C20	−20 (3)
C2—C1—C7—O1	−130.1 (7)	C17—O9—C20—C19	1.6 (9)
C6—C1—C7—O1	45.3 (9)	Zn2—O9—C20—C19	−170.6 (5)
Zn2^v^—O3—C8—O4	0.1 (6)	C21—C19—C20—O9	−17.1 (14)
Zn2^v^—O3—C8—C4	−175.0 (6)	C18—C19—C20—O9	11.8 (17)
Zn1^v^—O4—C8—O3	125.7 (6)	C29—O10—C22—C23	−94 (13)
Zn2^v^—O4—C8—O3	−0.1 (7)	C26—O10—C22—C23	−5 (2)
Zn1^v^—O4—C8—C4	−59.0 (8)	C25—O10—C22—C23	−28 (3)
Zn2^v^—O4—C8—C4	175.3 (5)	Zn1—O10—C22—C23	−164.3 (13)
Zn1^v^—O4—C8—Zn2^v^	125.8 (5)	O10—C22—C23—C24	18 (3)
C5—C4—C8—O3	73.2 (9)	C22—C23—C24—C25	−2 (2)
C3—C4—C8—O3	−113.0 (8)	C22—O10—C25—C24	27 (2)
C5—C4—C8—O4	−102.3 (7)	C29—O10—C25—C24	32 (2)
C3—C4—C8—O4	71.6 (9)	C26—O10—C25—C24	−64 (2)
Zn1^vi^—O6—C9—O5	−11.4 (8)	Zn1—O10—C25—C24	167.3 (13)
Zn1^vi^—O6—C9—C10	167.6 (4)	C23—C24—C25—O10	−15 (2)
Zn2—O5—C9—O6	−149.5 (5)	C22—O10—C26—C27	−44 (2)
Zn2—O5—C9—C10	31.5 (9)	C29—O10—C26—C27	−38 (2)
O6—C9—C10—C12^iii^	−106.3 (7)	C25—O10—C26—C27	53 (2)
O5—C9—C10—C12^iii^	72.7 (8)	Zn1—O10—C26—C27	114.9 (15)
O6—C9—C10—C11	73.4 (8)	O10—C26—C27—C28	32 (3)
O5—C9—C10—C11	−107.6 (7)	C26—C27—C28—C29	−14 (3)
C12^iii^—C10—C11—F5	−178.4 (6)	C22—O10—C29—C28	124 (15)
C9—C10—C11—F5	2.0 (9)	C26—O10—C29—C28	32 (3)
C12^iii^—C10—C11—C12	0.6 (10)	C25—O10—C29—C28	9 (3)
C9—C10—C11—C12	−179.1 (6)	Zn1—O10—C29—C28	−122.8 (17)
F5—C11—C12—F6	−0.7 (9)	C27—C28—C29—O10	−10 (3)
C10—C11—C12—F6	−179.7 (5)		

Symmetry codes: (i) −*x*+3/2, *y*+1/2, −*z*+1/2; (ii) *x*+1/2, −*y*+1/2, *z*+1/2; (iii) −*x*+1, −*y*, −*z*; (iv) −*x*+2, −*y*, −*z*; (v) *x*−1/2, −*y*+1/2, *z*−1/2; (vi) −*x*+3/2, *y*−1/2, −*z*+1/2.
